# Clinical Practice Consensus Statement 2025: Management of Hyperuricemia and Gout in Adolescents

**DOI:** 10.1111/1756-185x.70378

**Published:** 2025-07-22

**Authors:** Changgui Li, Jie Lu, Zhaohui Lyu, Haibing Chen, Benli Su, Bo Ban, Jianhua Mao, Ping Liu, Zhifeng Cheng, Jun Zhu, Naijun Wan, Xiaobo Chen, Chunxiu Gong, Peiyu Ye, Mingshu Sun, Wenyan Sun, Hui Zhang, Han Yin, Xiaoyun Jiang, Qing Wang, Yaolong Chen, Hsiao‐Yi Lin, Jie Mi, Jiajun Zhao

**Affiliations:** ^1^ Shandong Provincial Clinical Research Center for Immune Diseases and Gout The Affiliated Hospital of Qingdao University Qingdao China; ^2^ The Affiliated Xiang'an Hospital of Xiamen University Xiamen Fujian China; ^3^ The First Medical Center of Chinese PLA General Hospital Beijing China; ^4^ Shanghai Tenth People's Hospital Tongji University School of Medicine Shanghai China; ^5^ The Second Hospital of Dalian Medical University Dalian China; ^6^ The Affiliated Hospital of Jining Medical University Jining China; ^7^ Children Hospital of Zhejiang University School of Medicine & Liangzhu Laboratory Zhejiang University School of Medicine Hangzhou China; ^8^ The Second Affiliated Hospital School of Medicine, The Chinese University of Hong Kong, Shenzhen & Longgang District People's Hospital of Shenzhen Shenzhen China; ^9^ The Fourth Affiliated Hospital of Harbin Medical University Harbin China; ^10^ Shenzhen Municipal Bao'an District Hospital Shenzhen China; ^11^ Beijing Jishuitan Hospital Capital Medical University Beijing China; ^12^ Capital Center for Children's Health Capital Medical University Beijing China; ^13^ Center for Non‐Communicable Disease Management, Beijing Children's Hospital Capital Medical University, National Center for Children's Health Beijing China; ^14^ The Affiliated Zhongda Hospital of Southeast University Nanjing China; ^15^ The First Affiliated Hospital Sun Yat‐Sen University Guangzhou China; ^16^ Sino Japanese Friendship Hospital of Jilin University Changchun China; ^17^ Research Unit of Evidence‐Based Evaluation and Guidelines, Chinese Academy of Medical Sciences (2021RU017), School of Basic Medical Sciences, Lanzhou University Lanzhou China; ^18^ Division of Allergy Immunology and Rheumatology, Cheng‐Hsin General Hospital National Yang Ming Chiao Tung University Taibei Taiwan China; ^19^ Shandong Provincial Hospital Affiliated to Shandong First Medical University Jinan China

**Keywords:** adolescents, consensus, gout, hyperuricemia, management

## Abstract

**Background:**

In response to the increasing global prevalence of gout, there is a concerning shift towards a younger demographic, with China at the forefront of this trend. Hyperuricemia, a central factor in the pathogenesis of gout, is becoming increasingly common among adolescents, particularly males, and is associated with various health risks, including joint pain, CKD, metabolic disorders, and premature death. Despite the seriousness of this issue, there is a lack of specific guidelines addressing adolescent and hyperuricemia gout management.

**Methods:**

The working comprising 26 clinician pediatricians, rheumatologists, and endocrinologists, all experienced in the clinical presentation and management of gout and hyperuricemia, was convened to develop a consensus. A systematic literature search was conducted in PubMed, the Cochrane Library, and EMBASE published from 1 January 1960 to 31 May 2024. Two rounds of Delphi surveys for each recommendation were conducted among all group members via electronic questionnaire.

**Results:**

Adolescent‐onset gout is characterized by a pronounced genetic predisposition and distinct environmental influences, with a significant number of cases reporting a positive family history. We issued three consensus statements with five recommendations including the criteria, the urate‐lowering treatment, and flare therapy principles for hyperuricemia and gout in adolescents.

**Conclusions:**

This consensus statement comprehensively delves into the critical clinical challenges associated with gout and hyperuricemia in the adolescent population, emphasizing the pressing requirement for improved detection and management strategies to support a demographic that may be underserved by the healthcare system.

## 1 Introduction

In recent years, the prevalence of gout has increased globally, although documented cases among adolescents and young adults remain rare [1, 2]. A decade‐long retrospective study has highlighted a trend towards earlier onset of gout in China [3]. Hyperuricemia is not only an important precursor in the development of gout, but also its most significant risk factor [4]. The prevalence of hyperuricemia among Chinese adolescents, particularly males, has notably risen, with rates ranging from 26.6% to 42.3%, paralleling an increase in gout incidence [5, 6]. Adolescent (defined as 10–19 years old by World Health Organization)‐onset gout not only results in joint pain and deformities but also contributes to hepatic and renal dysfunction, dyslipidemia, and hypertension [7, 8], thereby exacerbating the progression of chronic kidney disease (CKD), precipitating cardiovascular disease, and increasing mortality risk [9, 10]. However, no guidelines or consensus specifically addressing the gout management in adolescents have been established so far.

Adolescent‐onset gout is characterized by a more pronounced genetic predisposition and unique environmental influences compared to its adult counterpart [5, 11, 12] (Table 1). Studies indicate that adolescent‐onset gout patients exhibit higher rates of obesity and genetic predisposition, but significantly lower rates of comorbidities such as hypertension, diabetes mellitus, renal disease, and alcohol consumption compared to adult gout [11, 13, 14, 15].

**TABLE 1:** The clinical features of adolescent‐onset gout.

Gout patients/Features	Adolescents	Adults
Onset age	10–19 years	> 19 years
Serum urate level	Higher, ~714 μmol/L	~540 μmol/L
Suffering joint	Finger joints, ankle joints; often polyarticular attacks	Metatarsophalangeal joints, knee joints, ankle joints
Flare	Experienced frequent flares, ~78.1%	~69%
Tophus	Fast progression, with an average of 1.5 years	With an average of 7.5 years
Family history of gout	A more pronounced genetic predisposition, ~38.7%	~15.4%
Genetics	Less documented; only *ABCG2, RCOR1*, etc.	More than hundreds of susceptible genes
Lifestyle	High‐fructose intake	Smoking, alcohol intake
Management	Poor; no specific guidelines or consensus have been established; only 17.9% of patients who met 2015 ACR/EULAR classification criteria for ULT initiated treatment and only 17.2% achieve ULT target (SU level of < 6.0 mg/dL)	Average; 2012 ACR guideline; 38% of patients who met guidelines for ULT initiated treatment and 23% achieve ULT target

A significantly higher incidence of a positive family history further underscores the critical role of genetic factors in the early onset of gout among adolescents [16]. Research has demonstrated that genetic factors can drive gout development even in the absence of traditional risk factors such as hypertension, diabetes, hypertriglyceridemia, hypercholesterolemia, and CKD [17]. Consequently, gout is recognized as a polygenic genetic disorder with a predominant genetic predisposition and an interaction between genetic and environmental factors. Recent studies have identified *ABCG2* dysfunction as a significant independent risk factor for adolescent‐onset hyperuricemia/gout [12, 18]. Furthermore, *SLC2A9* has been shown to exert a more pronounced effect on serum urate levels and hyperuricemia in children than in adults [19].

A recent study at a gout clinic in China revealed that over 80% of patients with adolescent‐onset gout met the 2015 ACR/EULAR gout classification criteria for initiating urate‐lowering therapy (ULT), while only 17.9% of patients initially received the recommended therapy [15]. Alarmingly, just 17.2% of patients undergoing ULT achieved the therapeutic target. This shortfall may be attributed to low adherence [20], suboptimal dosing of urate‐lowering medications [21], and inadequate medication management [20], as well as potential genetic resistance.

This consensus statement comprehensively addresses three pivotal clinical aspects inquiries pertaining to adolescent gout. Importantly, clinicians must recognize that adolescent‐onset gout is a potentially underserved demographic requiring targeted interventions and tailored management strategies.

## 2 Methods

A group comprising 26 clinician pediatricians, rheumatologists, and endocrinologists, all experienced in the clinical presentation and management of gout and hyperuricemia, was convened to develop a consensus. A set of questions was formulated to define the topics to be addressed by the literature review. These questions were subsequently reviewed by the consensus panel to ensure the inclusion of clinically pertinent topics in the investigation and management of gout and hyperuricemia in adolescents. This consensus has been officially registered on the International Practice Guidelines Registration Platform (http://www.guidelines‐registry.org), under the registration number: PREPARE‐2024CN1074.

A systematic literature search was conducted in PubMed, the Cochrane Library, and EMBASE published from 1 January 1960 to 31 May 2024. The search employed the following terms: (“Adolescents” OR “Adolescent” OR “Adolescence” OR “Teens” OR “Teen” OR “Teenagers” OR “Teenager” OR “Youth” OR “Youths” OR “young adult” OR “Juvenile” OR “Pediatric” OR “Pediatrics”) AND (“Hyperuricemia” OR “Urate” OR “uric acid” OR “Gout” OR “Gouty”). Titles and abstracts of all search results were reviewed, and the full texts were assessed if the publication was relevant to the question. The Grading of Recommendations Assessment, Development, and Evaluation (GRADE) system was used, incorporating evidence from systematic literature reviews, standardized evidence quality assessment, and expertise consensus. A multidisciplinary Expert Panel (EP) was assembled for clinical question proposals, incorporating clinical evidence and physicians' input, finalized seven key questions, which were deconstructed using the Patient/Population, Intervention, Comparison, Outcome (PICO) principle (Table 2). Two rounds of Delphi voting were conducted to iteratively revise and verify the clinical questions and recommendations based on existing evidence, the certainty and confidence, determining the strength of the recommendations via electronic questionnaire in July and September 2024, with effective response rates of 87.5% and 91.7%, respectively.

**TABLE 2:** Statement deconstruction with PICO method.

Specific statement	P (Patients)	I (Intervention)	C (Comparison)	O (Outcome)
I. Criteria for hyperuricemia and gout in adolescents	Patients with adolescent (defined as 10–19 years old by World Health Organization)‐onset hyperuricemia or gout	Refer to adults	Other standards	Serum urate (SU) levels, urate solubility, urate deposition, gouty tophi, bone erosion, etc.
II. Principles of urate‐lowering treatment for adolescent hyperuricemia and gout	Patients with adolescent (defined as 10–19 years old by World Health Organization)‐onset hyperuricemia or gout who require urate‐lowering treatment	Administer urate‐lowering medications such as febuxostat, allopurinol, benzbromarone, and uricase	No urate‐lowering medication used	The rate of achieving target SU levels, incidence of gout, incidence of new kidney stones, onset of new hypertension or progression of existing hypertension, occurrence and progression of other MetS components, and their effects on growth, development, and reproduction (height, weight, sperm quantity and quality, etc.)
III. Treatment principles for gout flares in adolescents	Patients with adolescent (defined as 10–19 years old by World Health Organization)‐onset hyperuricemia or gout who require acute gout flare treatment	Administer colchicine, NSAIDs, and glucocorticoids selection and priority	Other drugs control	Duration of pain, degree of pain, duration of illness, changes in renal function, components of metabolic syndrome, effects of cardiovascular and cerebrovascular diseases, drug side effects (effects on bones, organs, reproduction, etc.)

## 3 Results and Discussion

The consensus group formulated statements addressing seven key questions identified through a literature review, with three additional questions discussed solely within the text of the manuscript (Table 3).

**TABLE 3:** Summary of recommendations, recommendation strength, and evidence level.

Index	Recommendations	Strength of recommendations	Level of evidence	Agreement rate (disagree number)
Consensus statement I: Criteria for hyperuricemia and gout in adolescents	It is recommended that the criteria for hyperuricemia in adolescents (WHO defined 10–19 years old) refer to adult standards. Under normal dietary conditions, fasting blood urate level should be ≥ 420 μmol/L on two different days	2	C	100% (0)
	It is recommended that the classification criteria for adolescent gout refer to adult standards, using the 2015 ACR/EULAR classification criteria.	2	C	100% (0)
Consensus statement II: Principles of urate‐lowering treatment for adolescent hyperuricemia and gout	Adolescent patients with hyperuricemia and gout are advised to maintain a healthy lifestyle and have regular monitoring of their blood urate levels	Good Practice Statement		100% (0)
	Asymptomatic patients with hyperuricemia should receive lifestyle guidance and have regular monitoring of their blood urate levels; It is recommended to start ULT in the following cases: (1) Blood urate level remain ≥ 420 μmol/L after 3–6 months of lifestyle guidance, or (2) Blood urate level ≥ 540 μmol/L, or (3) Blood urate level ≥ 480 μmol/L with one or more of the following complications: obesity with at least one metabolic abnormality (hypertension, abnormal lipid metabolism, diabetes), renal dysfunction (CKD ≥ 2), kidney stones, or urate crystal deposition. For patients without comorbidities, it is recommended to control blood urate at < 420 μmol/L. For those with comorbidities, it is recommended to control blood urate at < 360 μmol/L (GPS); For adolescent gout patients, ULT should be initiated in the following cases: (1) Blood urate levels are ≥ 480 μmol/L without complications or (2) Blood urate levels are ≥ 420 μmol/L with complications, including hypertension, abnormal lipid metabolism, diabetes, obesity, renal function damage (CKD ≥ 2 stages), kidney stones, uric acid crystal deposition, gout stones, recurrent gout flares, chronic gouty arthritis. For adolescent gout patients, the target blood urate level is < 300 μmol/L (GPS). For adolescent patients requiring ULT, it is recommended to refer to the drug instructions for medication selection, and closely monitor adverse events during medication	2	C	83.9% (10)
Consensus statement III: Treatment principles for gout flares in adolescents	For adolescents with gout flares, it is recommended to initiate anti‐inflammatory and analgesic treatment as soon as possible. Pharmacological therapy may include short‐term oral administration of glucocorticoids (not exceeding 7 days), nonsteroidal anti‐inflammatory drugs (NSAIDs), and colchicine. During the treatment period, close monitoring should be paid to adverse events	2	C	88.7% (7)

### 3.1 Consensus Statement I: Criteria for Hyperuricemia and Gout in Adolescents

- It is recommended that the criteria for hyperuricemia in adolescents (WHO defined 10–19 years old) refer to adult standards. Under normal dietary conditions, fasting blood urate level should be ≥ 420 μmol/L on two different days (2C).
- It is recommended that the classification criteria for adolescent gout refer to adult standards, using the 2015 ACR/EULAR classification criteria (2C).

The Guidelines for the Definition of Diseases developed by the Working Group for the Development of International Guidelines emphasize eight key considerations for revising disease definitions [22]: (1) differences between new and previous definitions, (2) implications for disease incidence and prevalence, (3) rationale for revising definitions, (4)predictive capability, (5) precision and accuracy, (6) benefits, (7) harm, (8) ratio of net benefits and hazards.

Under physiological conditions characterized by a body temperature of 37.0°C and a pH of 7.4, urate has a solubility of 381 μmol/L (6.4 mg/dL) in the blood [4], with its saturation point of 420 μmol/L (7.0 mg/dL) [23]. Recent survey data indicate that the prevalence of hyperuricemia among Chinese adolescents exceeds 25%, with over 40% of adolescent males affected with the cut‐point of 7.0 mg/dL [5, 6]. This growing prevalence is a major contributor to the rising incidence of gout and the development of CKD in this demographic [3, 15]. Chronic hyperuricemia in adolescents is associated with severe renal dysfunction, accelerate CKD progression, and increase the risk of renal failure and mortality [7, 9, 10]. Evidence suggests that adolescent patients with serum urate levels ≥ 420 μmol/L (7.0 mg/dL) have a significant higher an all‐cause mortality rate (HR, 1.38; 95% CI: 1.13–1.69; *p* < 0.001), with strongly correlations to increased mortality from cardiovascular disease (HR, 5.0; 95% CI 1.79–13.94; *p* = 0.001) and kidney disease (HR, 11.71; 95% CI 3.13–43.78; *p* = 0.001) [9]. Approximately 36% of adolescent patients with serum urate levels ≥ 330 μmol/L (5.5 mg/dL) experience a ≥ 30% decline in eGFR within 5 years. Additionally, blood urate levels ≥ 450 μmol/L (7.5 mg/dL) kidney injury progression by 38% [10]. The administration of allopurinol to decrease urate levels significantly reduces blood pressure in adolescents with hyperuricemia and hypertension [24]. Lowering blood urate from 420 μmol/L (7.0 mg/dL) to 252 μmol/L (4.2 mg/dL) has been shown to reduce systolic blood pressure by 6.9 mmHg and diastolic blood pressure by 5.1 mmHg. Currently, there is insufficient evidence to establish hyperuricemia criteria specific to adolescents based on age and gender. Considering the maximum solubility of urate in blood, its documented harm to adolescents, the 2017 disease definition guidelines, and the 2015 ACR/EULAR gout classification criteria, this consensus temporarily recommends adopting adult standards for defining hyperuricemia in adolescents.

At present, large‐scale epidemiological studies on gout in adolescents are lacking globally. Adolescents exhibit a more pronounced genetic predisposition [5] for gout compared to adults, along with an elevated risk of kidney and cardiovascular complications and mortality. The lack of large‐scale and high‐level epidemiological evidence poses challenges to the diagnosis and prevention of gout in this population. Multiple cross‐sectional studies on adolescent gout refer to the adult classification standards [13, 15], specifically the 2015 ACR/EULAR gout classification criteria. Despite the lack of evidence specific to adolescents, the group agreed that these criteria are appropriate to classify gout in adolescents.

### 3.2 Consensus Statement II: Principles of Urate‐Lowering Treatment for Adolescent Hyperuricemia and Gout

- Adolescent patients with hyperuricemia and gout are advised to maintain a healthy lifestyle and have regular monitoring of their blood urate levels (Good Practice Statement, GPS).
- Asymptomatic patients with hyperuricemia should receive lifestyle guidance and have regular monitoring of their blood urate levels; It is recommended to start ULT in the following cases: (1) Blood urate level remain ≥ 420 μmol/L after 3–6 months of lifestyle guidance or (2) Blood urate level ≥ 540 μmol/L, or (3) Blood urate level ≥ 480 μmol/L with one or more of the following complications: obesity with at least one metabolic abnormality (hypertension, abnormal lipid metabolism, diabetes), renal dysfunction (CKD ≥ 2), kidney stones, or urate crystal deposition. For patients without comorbidities, it is recommended to control blood urate at < 420 μmol/L. For those with comorbidities, it is recommended to control blood urate at < 360 μmol/L (GPS); For adolescent gout patients, ULT should be initiated in the following cases: (1) Blood urate levels are ≥ 480 μmol/L without complications or (2) Blood urate levels are ≥ 420 μmol/L with complications, including hypertension, abnormal lipid metabolism, diabetes, obesity, renal function damage (CKD ≥ 2 stages), kidney stones, uric acid crystal deposition, gout stones, recurrent gout flares, chronic gouty arthritis. For adolescent gout patients, the target blood urate level is < 300 μmol/L (GPS). For adolescent patients requiring ULT, it is recommended to refer to the drug instructions for medication selection, and closely monitor adverse events during medication (2C). (*Drug instructions note: (1) Allopurinol: Exercise caution when prescribing to children under 14 years old, with dosage adjustments as necessary. Testing for HLA‐B *5801 testing is recommended before administration, and only HLA‐B5801‐negative patients should be treated under physician guidance and adult supervision. (2) Febuxostat: The safety and efficacy of patients under 18 years old are yet to be established. If necessary, medications should be administered under medical supervision and with parental oversight. (3) Benzbromarone: Not recommended for children under 14 years old. For patients over 14 with poor excretion through 24‐h urine uric acid testing, a dosage of 50–100 mg/day is recommended. During treatment, it is necessary to drink a large amount of water to increase urine volume. Regular urine pH monitoring is advised, and a citric acid mixture or sodium bicarbonate should be added to maintain a urine pH of 6.5–6.8).

Adolescence is a critical period for growth and development. For adolescents with hyperuricemia and gout, comprehensive care should prioritize balanced nutrition, appropriate exercise, adequate water intake, and limiting of fructose and high purine food intake [25, 26, 27]. However, there are no established guidelines or clear recommendations for the timing and targets of ULT in this population. A retrospective study [13](*N* = 111) in China reported the clinical characteristics and treatment outcomes for adolescent gout patients. The results showed that 37 patients received febuxostat 40 mg/day for 3 months after the acute phase, resulting in a significant median reduction in blood urate levels (3.6 mg/dL) and a notable decrease in gouty arthritis attacks (pre‐treatment: 176/679 patient‐months vs. post‐treatment: 14/493 patient‐months, *p* < 0.0001). Four cases of adverse reactions to febuxostat were recorded: three with transient transaminase elevation which returned to normal without interruption of treatment, and 1 with rhabdomyolysis while fully recovered after stopping febuxostat treatment for 10 days. Another retrospective cross‐sectional study [28](*N* = 696 277) in Japan explored urate‐lowering therapy in children and adolescents. It found that 35.1% (97/276) of patients with gout or asymptomatic hyperuricemia received urate‐lowering drugs, with increasing use by age, particularly in males. Among the 97 patients treated, 54 received treatment with febuxostat, 31 with allopurinol, 15 with benzbromarone, 6 with tobramstat, and 1 with probenecid. The application of urate‐lowering therapy varied among patients with comorbidities: 47.9% (46/96) of those with concomitant kidney disease, 41.3% (26/63) with concomitant cardiovascular disease, 40.0% (6/15) with concomitant Down syndrome, and 27.1% (32/118) with concomitant metabolic syndrome. For patients with kidney disease, febuxostat was prescribed more than twice as frequently as allopurinol (28:12). The medication possession ratio (MPR) for febuxostat and allopurinol were 70.1% and 76.7%, respectively, with median prescription durations of 256 days for febuxostat and 280 days for allopurinol. For patients aged 6–11, the prescribed dose of these two drugs were about half the adult dose, while for those aged 12–18, the prescribed dose were roughly equivalent to adult dosages. A 2012 randomized, double‐blind, placebo‐controlled trial [29] compared the effects of urate‐lowering drugs with placebo on blood pressure in adolescents aged 11–17 years with prehypertension and obesity. The results showed that patients treated with allopurinol (week 1: 100 mg bid; weeks 2–7: 200 mg bid), or probenecid (week 1: 250 mg bid.; weeks 2–7: 500 mg bid.) showed significant reductions in blood pressure without reported adverse events.

Adolescents face a heightened risk of hyperuricemia and gout compared to adults [15]. Consequently, urate‐lowering therapy may be equally, if not more, beneficial in adolescent patients as it is for adults. This consensus statement emphasizes the clinical characteristics of hyperuricemia and gout in adolescents, including: (1) Comorbidities: Greater emphasis on metabolic syndrome, renal dysfunction (CKD stage ≥ 2), and urate crystal deposition, with less focus on cardiovascular and cerebrovascular diseases. (2) Target setting: urate target value < 360 μmol/L for adolescent hyperuricemia and < 300 μmol/L for adolescent gout. (3) Clinical typing: All adolescent patients requiring ULT should undergo renal uric acid excretion typing to guide drug selection based on clinical classification. (4) Special notes: For adolescents requiring ULT, it is recommended to refer to the drug instructions. If off‐label use is required, the patient and their guardian must sign an informed consent form. Vigilant monitoring for adverse events throughout the treatment course is essential.

### 3.3 Consensus Statement III: Treatment Principles for Gout Flares in Adolescents

For adolescents with gout flares, it is recommended to initiate anti‐inflammatory and analgesic treatment as soon as possible. Pharmacological therapy may include short‐term oral administration of glucocorticoids (not exceeding 7 days), nonsteroidal anti‐inflammatory drugs (NSAIDs), and colchicine. During the treatment period, close monitoring should be paid to adverse events (2C).

Compared to adults, adolescent gout patients exhibit higher levels of blood urate and frequency of attacks, more affected sites, and higher pain scores [13, 15]. Long‐term recurrent attacks have a dual impact on the physical and psychological well‐being of adolescents, negatively affecting their academic and family relationships. Therefore, early prevention and prompt treatment of acute gout attacks are necessary in this population.

For adolescents, the safety of pharmacological interventions is of paramount importance. Currently, there are no comprehensive reports on the safety and efficacy of commonly used drugs, such as colchicine, NSAIDs, and glucocorticoids, in adolescent gout patients. A 2022 retrospective study [13](*N* = 111) in China reported that adolescent gout patients with acute attacks were treated with empirical therapies, including oral NSAIDs or colchicine, along with dietary control (dosage and administration not reported). An observational study [30](*N* = 122) on children aged 2–10 years examined the tolerability and safety of various oral steroids, with an average prednisone dose of 2 mg/kg/day administered for 3–5 days, showing good tolerability. A systematic review showed that colchicine doses for adolescents with Familial Mediterranean Fever could be increased to 2 mg/day based on adult dosages. However, no specific thresholds for non‐toxic, toxic, or lethal colchicine doses in children have been established. Reports indicate that the lowest lethal dose of colchicine ranges from 7 to 26 mg/day [31], and the youngest fatality due to excessive colchicine was a 12‐month‐old child. NSAIDs have demonstrated efficacy in treating adolescent idiopathic arthritis, although they are often associated with adverse effects, including diarrhea, headache, nausea, constipation, rash, dizziness, and abdominal pain [32]. Studies suggest that oral prednisone is equally effective as NSAIDs for treating acute gout but offers a better safety profile [33]. Prednisone is widely recognized as a safe and effective treatment for acute gout [34]. Oral corticosteroids were proven effective in diseases such as idiopathic arthritis in adolescents [35]. Compared with NSAIDs, glucocorticoids exhibit similar efficacy in pain relief during gout attacks, but are associated with significantly lower rates of gastrointestinal adverse events (such as indigestion, nausea, vomiting, or gastrointestinal bleeding) [36, 37] Additionally, glucocorticoids have a relatively smaller impact on renal function [38]. A systematic review (*N* = 1112) compared the efficacy and safety of nine NSAIDs in the treatment of adolescent idiopathic arthritis (JIA), showing that among the four nonsteroidal anti‐inflammatory drugs, celecoxib, rofecoxib, meloxicam, and naproxen, celecoxib (6 mg/kg, twice a day) is the most effective [39].

## 4 Novelty and Conclusion

Adolescents are in a critical period of growth and development, undergoing gradual physiological maturation and refinement. Compared to adults, hyperuricemia and gout pose greater risks to adolescents with distinct clinical features—higher serum uric acid levels, increased frequency of flares, broader anatomical involvement, and elevated pain intensity scores [13, 15]. Prolonged uncontrolled attacks impose dual physiological and psychological burdens on adolescents, adversely impacting academic performance and family well‐being. Consequently, early intervention is imperative in this specific population.

This consensus explicitly addresses ongoing organ maturation (renal, hepatic, and neuroendocrine systems), requiring stricter safety thresholds and modified treatment targets, while the adult consensus focuses on established physiology with emphasis on atherosclerotic risks, CKD (Stages 3–5), and tophaceous gout. It recommends that all adolescent patients requiring ULT should undergo renal uric acid excretion typing to guide drug selection based on clinical classification, as adolescent‐onset gout patients had a greater proportion of underexcretion type than adults [15]. Dietary control is an effective measure in ULT for both adolescents and adults, while it should not be as strict in adolescents as their growth and development need. Moreover, the lack of pharmacokinetic data for colchicine/NSAIDs and the unknown long‐term effects of urate‐lowering therapy on developing systems impel physicians to be more careful when treating adolescents, and informed consent and periodic monitoring are necessary.

This consensus statement firstly identifies significant gaps in the management of hyperuricemia and gout in adolescents, and proposes a research agenda to address these critical uncertainties. A deeper understanding of the management of hyperuricemia and gout in adolescents is urgently needed, alongside further insights into the safety profiles of treatments for gout flares and the requirements for flare prophylaxis in this population. Additional investigations are imperative to determine the safe dosing of urate‐lowering drugs. Furthermore, pre‐specified analyses of safety and efficacy based on age and body weight, employing standardized outcome measures, are vital for advancing evidence‐based practices.

## Author Contributions

Changgui Li conceived the idea and submitted the project with a contribution from Zhaohui Lvy to the Chinese Association of Endocrinology Executive Committee. Jiajun Zhao organized and chaired the meetings. Changgui Li was in charge of the whole process of the guideline development. Changgui Li, Jie Lu, and Jie Mi drafted the manuscript.

## Ethics Statement

The authors have nothing to report.

## Conflicts of Interest

The authors declare no conflicts of interest.

## Data Availability

The data that support the findings of this study are available from the corresponding author upon reasonable request.

## References

[1] R. Liu , C. Han , D. Wu , et al., “Prevalence of Hyperuricemia and Gout in Mainland China From 2000 to 2014: A Systematic Review and Meta‐Analysis,” BioMed Research International 2015 (2015): 762820. 26640795 10.1155/2015/762820 PMC4657091

[2] S. Ito , T. Torii , A. Nakajima , et al., “Prevalence of Gout and Asymptomatic Hyperuricemia in the Pediatric Population: A Cross‐Sectional Study of a Japanese Health Insurance Database,” BMC Pediatrics 20, no. 1 (2020): 481. 33059648 10.1186/s12887-020-02379-0 PMC7559194

[3] Q. Gao , X. Cheng , T. R. Merriman , et al., “Trends in the Manifestations of 9754 Gout Patients in a Chinese Clinical Center: A 10‐Year Observational Study,” Joint, Bone, Spine 88, no. 6 (2021): 105078. 32950704 10.1016/j.jbspin.2020.09.010

[4] N. Dalbeth , A. L. Gosling , A. Gaffo , and A. Abhishek , “Gout,” Lancet 397, no. 10287 (2021): 1843–1855. 33798500 10.1016/S0140-6736(21)00569-9

[5] J. Lu , W. Sun , L. Cui , et al., “A Cross‐Sectional Study on Uric Acid Levels Among Chinese Adolescents,” Pediatric Nephrology 35, no. 3 (2020): 441–446. 31811538 10.1007/s00467-019-04357-w PMC6968984

[6] J. Rao , P. Ye , J. Lu , et al., “Prevalence and Related Factors of Hyperuricaemia in Chinese Children and Adolescents: A Pooled Analysis of 11 Population‐Based Studies,” Annals of Medicine 54, no. 1 (2022): 1608–1615. 35695553 10.1080/07853890.2022.2083670 PMC9225777

[7] C. C. Lu , S. K. Wu , H. Y. Chen , W. S. Chung , M. C. Lee , and C. J. Yeh , “Clinical Characteristics of and Relationship Between Metabolic Components and Renal Function Among Patients With Early‐Onset Juvenile Tophaceous Gout,” Journal of Rheumatology 41, no. 9 (2014): 1878–1883. 25086077 10.3899/jrheum.131240

[8] M. Krzystek‐Korpacka , E. Patryn , I. Kustrzeba‐Wojcicka , J. Chrzanowska , A. Gamian , and A. Noczynska , “Gender‐Specific Association of Serum Uric Acid With Metabolic Syndrome and Its Components in Juvenile Obesity,” Clinical Chemistry and Laboratory Medicine 49, no. 1 (2011): 129–136. 20961193 10.1515/CCLM.2011.011

[9] S. H. Hsia , I. J. Chou , C. F. Kuo , et al., “Survival Impact of Serum Uric Acid Levels in Children and Adolescents,” Rheumatology International 33, no. 11 (2013): 2797–2802. 23817870 10.1007/s00296-013-2808-y

[10] K. E. Rodenbach , M. F. Schneider , S. L. Furth , et al., “Hyperuricemia and Progression of CKD in Children and Adolescents: The Chronic Kidney Disease in Children (CKiD) Cohort Study,” American Journal of Kidney Diseases 66, no. 6 (2015): 984–992. 26209544 10.1053/j.ajkd.2015.06.015 PMC4658318

[11] S. Y. Chen and M. L. Shen , “Juvenile Gout in Taiwan Associated With Family History and Overweight,” Journal of Rheumatology 34, no. 11 (2007): 2308–2311. 17937457

[12] B. Stiburkova , K. Pavelcova , M. Pavlikova , P. Ješina , and K. Pavelka , “The Impact of Dysfunctional Variants of ABCG2 on Hyperuricemia and Gout in Pediatric‐Onset Patients,” Arthritis Research & Therapy 21 (2019): 77. 30894219 10.1186/s13075-019-1860-8 PMC6425717

[13] S. Zheng , P. Y. Lee , Y. Huang , et al., “Clinical Characteristics of Juvenile Gout and Treatment Response to Febuxostat,” Annals of the Rheumatic Diseases 81, no. 4 (2022): 599–600. 34930759 10.1136/annrheumdis-2021-221762

[14] H. Yamanaka , “Gout and Hyperuricemia in Young People,” Current Opinion in Rheumatology 23, no. 2 (2011): 156–160. 21169841 10.1097/BOR.0b013e3283432d35

[15] Y. Li , T. R. Merriman , H. Chen , et al., “Clinical Characteristics of Adolescent‐Onset Gout in Chinese: A Hospital‐Based Cross‐Sectional Study,” Seminars in Arthritis and Rheumatism 65 (2024): 152405. 38335695 10.1016/j.semarthrit.2024.152405

[16] A. C. Ji , Y. Sui , X. M. Xue , et al., “Novel Genetic Loci in Early‐Onset Gout Derived From Whole‐Genome Sequencing of an Adolescent Gout Cohort,” Arthritis and Rheumatology 77 (2024): 107–115. 39118347 10.1002/art.42969

[17] H. Matsuo , K. Yamamoto , H. Nakaoka , et al., “Genome‐Wide Association Study of Clinically Defined Gout Identifies Multiple Risk Loci and Its Association With Clinical Subtypes,” Annals of the Rheumatic Diseases 75, no. 4 (2016): 652–659. 25646370 10.1136/annrheumdis-2014-206191 PMC4819613

[18] Y. Toyoda , K. Pavelcová , J. Bohatá , et al., “Identification of Two Dysfunctional Variants in the ABCG2 Urate Transporter Associated With Pediatric‐Onset of Familial Hyperuricemia and Early‐Onset Gout,” International Journal of Molecular Sciences 22, no. 4 (2021): 1935. 33669292 10.3390/ijms22041935 PMC7920026

[19] B. Rivera‐Paredez , L. MACíAS‐KAUFFER , J. C. Fernandez‐Lopez , et al., “Influence of Genetic and Non‐Genetic Risk Factors for Serum Uric Acid Levels and Hyperuricemia in Mexicans,” Nutrients 11, no. 6 (2019): 1336. 31207883 10.3390/nu11061336 PMC6627998

[20] S. Hu , R. Terkeltaub , M. Sun , et al., “Palpable Tophi and More Comorbidities Associated With Adherence to Urate‐Lowering Medical Therapy in a Chinese Gout Cohort,” Joint, Bone, Spine 89, no. 6 (2022): 105435. 35777552 10.1016/j.jbspin.2022.105435

[21] L. K. Stamp , T. R. Merriman , M. L. Barclay , et al., “Impaired Response or Insufficient Dosage?‐Examining the Potential Causes of “Inadequate Response” to Allopurinol in the Treatment of Gout,” Seminars in Arthritis and Rheumatism 44, no. 2 (2014): 170–174. 24925693 10.1016/j.semarthrit.2014.05.007 PMC4225179

[22] J. Doust , P. O. Vandvik , A. Qaseem , et al., “Guidance for Modifying the Definition of Diseases A Checklist,” JAMA Internal Medicine 177, no. 7 (2017): 1020–1025. 28505266 10.1001/jamainternmed.2017.1302

[23] T. Neogi , T. L. Jansen , N. Dalbeth , et al., “Gout Classification Criteria: An American College of Rheumatology/European League Against Rheumatism Collaborative Initiative,” Arthritis and Rheumatology 67, no. 10 (2015): 2557–2568. 26352873 10.1002/art.39254 PMC4566153

[24] D. I. Feig , B. Soletsky , and R. J. Johnson , “Effect of Allopurinol on Blood Pressure of Adolescents With Newly Diagnosed Essential Hypertension: A Randomized Trial,” JAMA 300, no. 8 (2008): 924–932. 18728266 10.1001/jama.300.8.924 PMC2684336

[25] F. Chen , K. Huang , Q. Long , et al., “Comparative Dietary Effectiveness of a Modified Government‐Recommended Diet With Avoidance of Ultra‐Processed Foods on Weight and Metabolic Management in Children and Adolescents: An Open‐Label, Randomized Study,” Asia Pacific Journal of Clinical Nutrition 31, no. 2 (2022): 282–293. 35766564 10.6133/apjcn.202206_31(2).0014

[26] S. Chiu , P. Siri‐Tarino , N. Bergeron , J. H. Suh , and R. M. Krauss , “A Randomized Study of the Effect of Replacing Sugar‐Sweetened Soda by Reduced Fat Milk on Cardiometabolic Health in Male Adolescent Soda Drinkers,” Nutrients 12, no. 2 (2020): 405. 32033078 10.3390/nu12020405 PMC7071288

[27] K. S. Bobridge , G. L. Haines , T. A. Mori , et al., “Dietary Fructose in Relation to Blood Pressure and Serum Uric Acid in Adolescent Boys and Girls,” Journal of Human Hypertension 27, no. 4 (2013): 217–224. 22971754 10.1038/jhh.2012.36

[28] M. Honda , H. Horiuchi , T. Torii , et al., “Urate‐Lowering Therapy for Gout and Asymptomatic Hyperuricemia in the Pediatric Population: A Cross‐Sectional Study of a Japanese Health Insurance Database,” BMC Pediatrics 21, no. 1 (2021): 581. 34922491 10.1186/s12887-021-03051-x PMC8684120

[29] B. Soletsky and D. I. Feig , “Uric Acid Reduction Rectifies Prehypertension in Obese Adolescents,” Hypertension 60, no. 5 (2012): 1148–1156. 23006736 10.1161/HYPERTENSIONAHA.112.196980

[30] F. Aljebab , M. Alanazi , I. Choonara , et al., “Observational Study on the Palatability and Tolerability of Oral Prednisolone and Oral Dexamethasone in Children in Saudi Arabia and the UK,” Archives of Disease in Childhood 103, no. 1 (2018): 83–88. 28735259 10.1136/archdischild-2017-312697 PMC5754874

[31] Y. Finkelstein , S. E. Aks , J. R. Hutson , et al., “Colchicine Poisoning: The Dark Side of an Ancient Drug,” Clinical Toxicology 48, no. 5 (2010): 407–414. 20586571 10.3109/15563650.2010.495348

[32] J. Yu , H. M. Lu , J. Zhou , et al., “Oral Prednisolone Versus Non‐Steroidal Anti‐Inflammatory Drugs in the Treatment of Acute Gout: A Meta‐Analysis of Randomized Controlled Trials,” Inflammopharmacology 26, no. 3 (2018): 717–723. 29357007 10.1007/s10787-018-0442-8

[33] C. Eccleston , T. E. Cooper , E. Fisher , B. Anderson , and N. M. Wilkinson , “Non‐Steroidal Anti‐Inflammatory Drugs (NSAIDs) for Chronic Non‐Cancer Pain in Children and Adolescents,” Cochrane Database of Systemic Review 8, no. 8 (2017): CD012537, 10.1002/14651858.cd012537.pub2. PMC6460508 28770976

[34] T. H. Rainer , C. H. Cheng , H. J. E. M. Janssens , et al., “Oral Prednisolone in the Treatment of Acute Gout A Pragmatic, Multicenter, Double‐Blind, Randomized Trial,” Annals of Internal Medicine 164, no. 7 (2016): 464–471. 26903390 10.7326/M14-2070

[35] K. J. Murray and D. J. Lovell , “Advanced Therapy for Juvenile Arthritis,” Best Practice Research 16, no. 3 (2002): 361–378. 12387805

[36] C. A. Billy , R. T. Lim , M. Ruospo , S. C. Palmer , and G. F. M. Strippoli , “Corticosteroid or Nonsteroidal Antiinflammatory Drugs for the Treatment of Acute Gout: A Systematic Review of Randomized Controlled Trials,” Journal of Rheumatology 45, no. 1 (2018): 128–136. 28765243 10.3899/jrheum.170137

[37] M. D. Wechalekar , O. Vinik , J. H. Y. Moi , et al., “The Efficacy and Safety of Treatments for Acute Gout: Results From a Series of Systematic Literature Reviews Including Cochrane Reviews on Intraarticular Glucocorticoids, Colchicine, Nonsteroidal Antiinflammatory Drugs, and Interleukin‐1 Inhibitors,” Journal of Rheumatology 41 (2014): 15–25. 25180124 10.3899/jrheum.140458

[38] A. Faggiano , R. Pivonello , D. Melis , et al., “Nephrolithiasis in Cushing's Disease: Prevalence, Etiopathogenesis, and Modification After Disease Cure,” Journal of Clinical Endocrinology & Metabolism 88, no. 5 (2003): 2076–2080. 12727957 10.1210/jc.2002-021494

[39] C. L. Shi , Y. Zhang , Z. Y. Zhang , J. Zhou , and X. M. Tang , “Comparative Efficacy and Safety of Non‐Steroidal Anti‐Inflammatory Drugs in Patients With Juvenile Idiopathic Arthritis: A Systematic Review and Network Meta‐Analysis,” Indian Pediatrics 58, no. 2 (2021): 162–168. 33632948

